# Outcome reporting in therapeutic mammaplasty: a systematic review

**DOI:** 10.1093/bjsopen/zrab126

**Published:** 2021-12-11

**Authors:** Alice Lee, Richard M Kwasnicki, Hasaan Khan, Yasmin Grant, Abigail Chan, Angela E E Fanshawe, Daniel R Leff

**Affiliations:** 1 Department of Surgery and Cancer, Imperial College London, London, UK; 2 Faculty of Medicine, Imperial College London, London, UK; 3 Department of BioSurgery, Imperial College London, London, UK; 4 Department of Breast Surgery, Charing Cross Hospital, Imperial College NHS Trust, London, UK

## Abstract

**Background:**

Therapeutic mammaplasty (TM) is an oncological procedure which combines tumour resection with breast reduction and mastopexy techniques. Previous systematic reviews have demonstrated the oncological safety of TM but reporting of critically important outcomes, such as quality of life, aesthetic and functional outcomes, are limited, piecemeal or inconsistent. This systematic review aimed to identify all outcomes reported in clinical studies of TM to facilitate development of a core outcome set.

**Methods:**

Medline, EMBASE, CINAHL and Web of Science were searched from inception to 5 August 2020. Included studies reported clinical outcomes following TM for adult women. Two authors screened articles independently for eligibility. Data were extracted regarding the outcome definition and classification type (for example, oncological, quality of life, etc.), time of outcome reporting and measurement tools.

**Results:**

Of 5709 de-duplicated records, 148 were included in the narrative synthesis. The majority of studies (*n* = 102, 68.9 per cent) reported measures of survival and/or recurrence; approximately three-quarters (*n* = 75, 73.5 per cent) had less than 5 years follow-up. Aesthetic outcome was reported in half of studies (*n* = 75, 50.7 per cent) using mainly subjective, non-validated measurement tools. The time point at which aesthetic assessment was conducted was highly variable, and only defined in 48 (64.0 per cent) studies and none included a preoperative baseline for comparison. Few studies reported quality of life (*n* = 30, 20.3 per cent), functional outcomes (*n* = 5, 3.4 per cent) or resource use (*n* = 28, 18.9 per cent).

**Conclusion:**

Given the oncological equivalence of TM and mastectomy, treatment decisions are often driven by aesthetic and functional outcomes, which are infrequently and inconsistently reported with non-validated measurement tools.

## Teaser

Given the oncological equivalence of therapeutic mammaplasty and mastectomy, treatment decisions are often driven by aesthetic and functional outcomes, which should have a robust evidence base. However, this review has shown such outcomes are infrequently and inconsistently reported with non-validated measurement tools. A core outcome set will enable standardization of reporting and synthesis of high-quality evidence to guide clinical decision-making and patient selection.

## Introduction

Therapeutic mammaplasty (TM) is an oncological procedure which aims to combine tumour resection with breast reduction and mastopexy techniques^1^. TM can facilitate breast-conserving surgery (BCS) in large tumour : breast volume ratio^2^ to avoid mastectomy^3^ safely and improve cosmesis in cases where standard BCS would otherwise yield poor outcome^4^. Other advantages of TM include fewer radiotherapy-related side effects in large-breasted women^4^^,^^5^ and alleviation of allied symptoms associated with macromastia^4^. Previous systematic reviews suggest TM is oncologically safe^2^^,^^4^^,^^6^, but there is inconsistent reporting of quality-of-life (QOL), aesthetic and functional outcomes, with numerous (often non-validated) measurement tools^5^^,^^7^^,^^8^. Furthermore, available outcome-measurement tools are likely to expand with increasing use of technology-based aesthetic and functional assessment^9–11^.

BCS is demonstrably safe when compared with mastectomy^12^, although TM is often performed to extend the boundaries of standard BCS and the tumours resected using this technique may therefore be larger than those included in BCS/mastectomy comparisons. This means that treatment decisions are often driven by aesthetic and patient-reported outcomes, which should have a robust evidence base. These outcomes are likely to differ on an individual patient level, but very little research has been done to explore patients’ treatment priorities^13^. Surgical morbidity, relating to postoperative complications, and delay to adjuvant therapy are also important factors, although recent, large prospective studies are reassuring^14^^,^^15^. Improving the quality and homogeneity of outcome measurement and reporting in TM is therefore an urgent priority, in order to facilitate high-quality meta-analyses and optimize patient selection. Standardization of outcome reporting could be achieved through development of a core outcome set (COS), which describes the minimum number of outcomes to be reported across all trials of one healthcare domain^16^. A COS is available for reconstructive breast surgery^17^, however this focused mainly on post-mastectomy reconstruction (only 10 per cent of patient stakeholders had undergone TM) and some outcomes included in the final COS are irrelevant to the TM population (such as implant-related complications). Moreover, there is reason to hypothesize that TM patients may evaluate and prioritize their treatment outcomes differently from patients undergoing other forms of breast reconstruction. For example, improved functional outcomes associated with breast reduction techniques and avoidance of mastectomy may drive treatment decisions significantly^4^^,^^14^^,^^18^.

A prerequisite of COS development is a comprehensive review of all available outcomes and outcome measures, which are then refined using consensus methodology into a final ‘set’. The primary objective of this review was to characterize the clinical, aesthetic, QOL and functional outcomes, as well as resource use, reported in clinical studies of TM. This includes any variation in outcome definitions, the measurement tools used and whether these are validated. The secondary objective was to identify variation in the timing of outcome measurement. The overall aim was to facilitate the development of a COS^19^ and to summarize current methods of outcome measurement, with a view to informing technological applications in the field.

## Methods

This systematic review adheres to a prespecified protocol and the PRISMA statement^20^. The protocol is available on PROSPERO (available from: https://www.crd.york.ac.uk/prospero/display_record.php?RecordID=200365) and has been peer-reviewed and published^18^.

### Identification of studies

This systematic review included clinical studies of adult, female participants who underwent TM as primary treatment for breast carcinoma or carcinoma *in situ*. For the purposes of the review, TM was defined as the use of oncoplastic reduction or mastopexy techniques, including removal of the skin envelope and/or nipple if indicated, to treat preinvasive or invasive breast cancer with BCS^21^. This correlates to level I–II oncoplastic breast surgery^22^. Inclusion and exclusion criteria are highlighted in *Table S1*.

All studies which reported patient outcomes following TM were included. Outcomes were extracted under various categories (clinical, aesthetic, QOL/patient-reported, functional or resource use), prior to being formally classified into domains.

The following electronic databases were searched from inception to 5 August 2020: OVID Medline, EMBASE, CINAHL and Web of Science. The reference lists of included studies were hand-searched for relevant articles. Outcomes generated from the review were also cross-referenced with those reported in the Oncoplastic Breast Reconstruction Guidelines for Best Practice co-produced by the Association of Breast Surgery and British Association of Plastic Reconstructive and Aesthetic Surgeons^23^^,^^24^.

A search string was developed to identify relevant papers including key search terms and relevant medical subject headings. An example search string for OVID Medline is shown in *Table S2*^18^. Validated study design filters for clinical trials, cohort studies and case–control studies^25^^,^^26^ were used to focus the search and manage screening numbers.

### Study selection process

Search results were de-duplicated and screened using Covidence software (Veritas Health Innovation, Melbourne, Australia; version 2103). Articles were screened in two stages (title and abstract; full text) by two independent reviewers (combinations of A.L., H.K., Y.G., A.C. and A.F.) against prespecified inclusion and exclusion criteria.

### Quality assessment

The aim of the review was to generate a comprehensive list of reported outcomes and outcome measures, regardless of methodological quality; hence, risk-of-bias assessment was not performed.

### Data extraction

Data were extracted using a piloted data extraction form (Microsoft Excel, version 16.46; Microsoft) developed for the purposes of the review (available on request). For each included study the following details were extracted: study design, population size and average age, average follow-up time, TM procedure (including skin-incision pattern) and inclusion within the cohort of symmetrization procedures and (neo)adjuvant radiotherapy. Outcomes were extracted across various categories including clinical, aesthetic, QOL/patient-reported and functional outcomes, as well as resource use (for example, duration of stay). Certain QOL outcome measures additionally included items covering aesthetic and functional outcomes; this is indicated in the text where relevant.

Extracted information included the outcome definition, method of outcome measurement, validation of the outcome in an oncoplastic population and time point of measurement. For aesthetic, functional and QOL outcomes, we recorded whether these were patient- or clinician-reported (or both), and if clinician-reported, whether the clinician was directly involved in care provision.

### Data synthesis

Extracted outcomes were grouped into domains according to an author-generated ontological framework^19^, adapted from a similar COS development project which focused mainly on post-mastectomy reconstruction^17^, to suit the characteristics of the extracted data. The data were then described narratively to characterize any variation in outcome definitions and measurement (primary outcomes) and the timing of outcome measurement (secondary outcome).

## Results

**Fig. 1:**
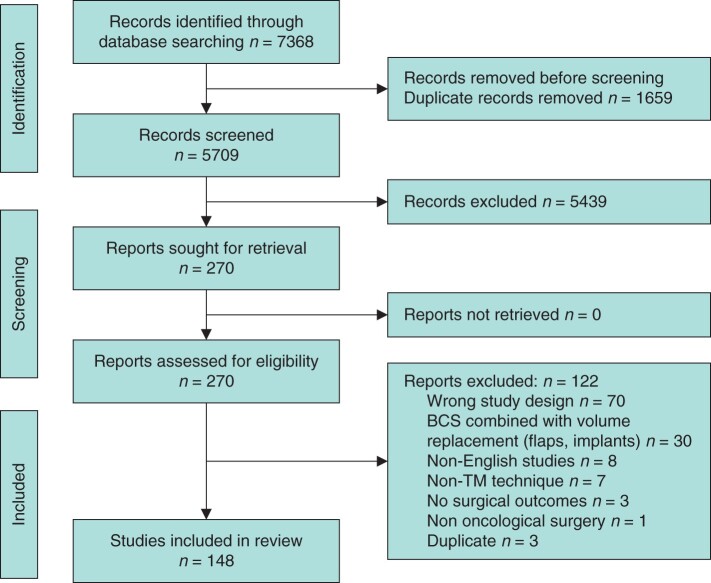
PRISMA flow chart BCS, breast-conserving surgery; TM, therapeutic mammaplasty.

Literature searches returned a total of 5709 de-duplicated articles, of which 5439 were excluded at the title and abstract stage. Of the 270 full-text articles assessed for eligibility, 122 were excluded, leaving 148 studies for narrative synthesis (*Fig. 1*; *Table S3*).

### Study characteristics

The majority of studies were retrospective cohort in design (*n* = 84, 56.8 per cent), included multiple skin-incision patterns and included patients who underwent contralateral symmetrization procedures and (neo)adjuvant radiotherapy (*Table 1*). Over half (*n* = 93, 62.8 per cent) of included studies had fewer than 100 participants (range 5–1024). The duration of follow-up ranged from 2 months to 10 years (median 32 months).

**Table 1:** Characteristics of included studies

Characteristics of included studies	Studies
Design	
Prospective	53 (35.8)
Retrospective	88 (59.5)
Unclear	7 (4.7)
Study type	
Cohort	143 (96.6)
Case–control	2 (1.4)
Case series	3 (2.0)
Skin incision used	
Wise and modified Wise pattern	18 (12.2)
Periareolar/circumareolar with skin excision (round block, Benelli, racquet)	4 (2.7)
Vertical scar	4 (2.7)
Multiple	83 (56.1)
Other	18 (12.2)
Contralateral symmetrization procedures included in cohort	101 (68.2)
Neoadjuvant or adjuvant radiotherapy included in cohort	134 (90.5)

Values in parentheses are percentages.

### Clinical outcomes

Clinical outcomes following TM were classified into three domains: oncological safety, surgical morbidity and detection of contralateral breast carcinoma or carcinoma *in situ* (*Table 2*).

**Table 2:** Clinical outcome domains

Domain	Outcomes
Oncological safety	Overall survival or mortality rate
	Breast-cancer-specific survival or mortality rate
	Disease- or progression-free survival
	Locoregional recurrence
	Distant recurrence/metastasis
	Reintervention (surgical and/or radiotherapy) for close or involved margins
Surgical morbidity	Surgical complications*
	Delay to adjuvant therapy
	Duration of drain insertion
	Further investigation for irregular breast symptoms after operation
For symmetrization procedures: detection of contralateral breast carcinoma or carcinoma *in situ*	

* A complete list of reported complications can be found in *Table S5*.

In the main, studies (*n* = 102, 68.9 per cent) reported one or more long-term oncological safety outcome, most frequently locoregional recurrence (*Table S4*). The follow-up period for these outcomes varied substantially; the majority (*n* = 75, 73.5 per cent) had follow-up times of less than 5 years. Almost all studies (*n* = 135, 91.2 per cent) reported margin status or the need for reintervention for oncological reasons (margin re-excision, completion mastectomy or additional radiotherapy boost). Three studies (2.0 per cent) presented these data as ability to achieve successful breast conservation^14^^,^^27^^,^^28^.

Most studies (*n* = 117, 79.1 per cent) reported surgical complications (*Table S5*). One study reported complications from a previously validated list (National Surgical Quality Improvement Program)^29^. A minority of studies classified complications according to morbidity, as ‘major’ or ‘minor’ (*n* = 14, 9.5 per cent)^1^^,^^14^^,^^28^^,^^30–38^ although definitions of ‘major’ varied, for example, necessitating surgical management or readmission. Only four (2.7 per cent) studies used the validated Clavien–Dindo classification^39–42^. Certain studies, which did not classify complications formally, did report complications requiring reoperation or readmission separately (*n* = 19, 12.8 per cent)^15^^,^^32^^,^^43–59^. Most studies (*n* = 95, 81.2 per cent) did not clarify the measurement period for postoperative complications. Where postoperative time points were specified they varied substantially, for example, within 30 days (*n* = 6)^15^^,^^29^^,^^42^^,^^59–61^, to 6 weeks (*n* = 1)^62^ and/or 6 months (*n* = 1)^57^. Certain studies classified complications as ‘immediate’ and/or ‘early’ and/or ‘late’ (*n* = 13), but then failed to define the temporal cut-offs. Where ‘late’ was defined it varied from as little as 14 days (*n* = 1)^54^ to 2 months (*n* = 4)^38^^,^^53^^,^^63^^,^^64^ or as long as 6 months (*n* = 2)^65^^,^^66^.

Less than one-third of included studies (*n* = 40, 27.0 per cent) reported delays or time to receive adjuvant therapy. The majority (*n* = 29, 72.5 per cent) did not define ‘delay’, and reported number of days/weeks until radiotherapy, chemotherapy or first adjuvant treatment. A minority of studies (*n* = 10, 25.0 per cent) defined delays to adjuvant therapy with varying temporal cut-offs, for example less than or equal to 4 weeks (*n* = 2)^15^^,^^48^, 6 weeks (*n* = 4)^8^^,^^32^^,^^41^^,^^67^ and 8 weeks (*n* = 2)^35^^,^^68^ after operation. Two studies differentiated the cut-off for chemotherapy and radiotherapy as 6 and 8 weeks after surgery, respectively^52^^,^^69^.

Two studies (1.4 per cent) reported duration of drain insertion^32^^,^^70^ and five studies (3.4 per cent) reported additional service use (non-routine imaging, tissue sampling) to investigate postoperative breast symptoms^53^^,^^71–74^.

Regarding symmetrization procedures, histological evaluation for contralateral occult malignancy was explicitly reported in 23 studies (15.5 per cent)^38^^,^^50^^,^^52^^,^^53^^,^^55^^,^^63^^,^^65^^,^^72^^,^^75–89^.

### Aesthetic outcomes

A total of 75 (50.7 per cent) studies reported aesthetic outcomes after TM (full list in *Table 3*). An additional six studies reported patient-reported outcome measures (PROMs), which included items assessing cosmesis (described in detail in the section below). All 75 studies used subjective aesthetic assessments; six studies also used objective methods (Breast Cancer Conservative Treatment. Cosmetic results (BCCT.core) software^40^^,^^45^^,^^78^^,^^90^^,^^91^ or breast symmetry index^92^).

**Table 3:** Aesthetic outcome domains

Domains	Outcomes
**Nipple–areola complex**	Shape Colour Sensation Position on the breast mound
**Breast**	Size Shape Symmetry Irradiation skin changes Projection Correction of ptosis Mobility on chest wall Consistency Inframammary fold Scars Bra fitting* Appearance clothed and unclothed* Overall appearance of breast Overall comparison before and after surgery
**Reoperation for cosmesis†**	

* These outcomes also feature in the quality-of-life section.

† This includes elective operations offered for cosmetic defects and not early surgical complications (such as skin necrosis).

Numeric or qualitative scoring systems were most commonly used for subjective assessment (*n* = 72, 96.0 per cent), based on patient self-assessment or clinical assessment (*Table 4*). In 27 studies (36.0 per cent)^31^^,^^40^^,^^42^^,^^65^^,^^66^^,^^68^^,^^73^^,^^85^^,^^86^^,^^88^^,^^90^^,^^93–108^, subjective aesthetic outcome assessment utilized two-dimensional digital patient photographs, where specified. In four studies^74^^,^^95^^,^^109^^,^^110^, patients were questioned ‘informally’, or the methodology was unclear. Only 19 studies used previously validated or published assessment tools^42^^,^^45^^,^^47^^,^^54^^,^^63^^,^^73^^,^^81^^,^^90^^,^^92–94^^,^^97^^,^^98^^,^^111–116^. One study compared the results of their own institutional aesthetic questionnaire with the Breast Cancer Treatment Outcomes Scale and found a significant correlation^117^.

**Table 4:** Aesthetic outcome measures

Aesthetic outcome measures	Studies*
Subjective	
Harris scale	6 (8.0)
ABNSW (assessing asymmetry, breast shape, nipple shape, skin condition and wound scar)	1 (1.3)
Regnault and Bostwick classification	1 (1.3)
Garbay criteria	1 (1.3)
Score system previously published or adapted from published material	11 (14.7)
Non-validated score system or questionnaire	50 (66.7)
Informal patient questioning or interview	2 (2.7)
Unclear	2 (2.7)
Objective	
BCCT.core software	5 (6.7)
Breast symmetry index	1 (1.3)
Reoperation for cosmetic reasons	25 (33.3)

Values in parentheses are percentages.

*Some studies used more than one outcome measure. A complete list of aesthetic outcomes can be found in *Table 3*. ABNSW, assessing asymmetry, breast shape, nipple shape, skin condition and wound scar.

Aesthetic outcome was assessed by the patient and clinician (*n* = 25, 33.3 per cent)^31^^,^^36^^,^^38^^,^^40^^,^^42^^,^^45^^,^^58^^,^^65^^,^^66^^,^^70^^,^^76^^,^^88^^,^^91–93^^,^^102^^,^^103^^,^^108^^,^^111^^,^^112^^,^^116^^,^^118–121^, clinician only (*n* = 25, 33.3 per cent)^39^^,^^63^^,^^68^^,^^73^^,^^78^^,^^79^^,^^81^^,^^85^^,^^96^^,^^97^^,^^99–101^^,^^107^^,^^113^^,^^122–126^, patient only (*n* = 22, 29.3 per cent)^2^^,^^47^^,^^49^^,^^54^^,^^64^^,^^67^^,^^72^^,^^74^^,^^89^^,^^94^^,^^109^^,^^110^^,^^114^^,^^117^^,^^127–135^ or was unclear (*n* = 2, 2.7 per cent)^95^^,^^136^. Very few studies (*n* = 4) included non-medical staff in aesthetic rating panels^63^^,^^73^^,^^86^^,^^108^. Where clinicians assessed aesthetic outcome, they were stated explicitly to be independent of care provision in 14 of 50 studies^31^^,^^42^^,^^45^^,^^76^^,^^97^^,^^99^^,^^100^^,^^106^^,^^112^^,^^113^^,^^115^^,^^116^^,^^122^^,^^123^. Similarly, few studies assessed correlation between aesthetic evaluation by clinicians and/or patient satisfaction and/or BCCT.core software^40^^,^^45^^,^^85^. Santos and colleagues reported poor concordance in aesthetic result evaluated by a patient questionnaire, specialists (Garbay criteria) and BCCT.core software^45^. Similarly, Egro and co-workers found no correlation between clinician-rated aesthetic outcome (7-point Likert scale) and patient satisfaction (BREAST-Q)^85^. In contrast, Matrai and colleagues found a positive correlation between BCCT.core software results and patient satisfaction on the BREAST-Q (psychosocial and physical wellbeing (chest) domains)^40^. These differences may be explained by the varying aesthetic scales and patient questionnaires used^40^^,^^45^^,^^85^, variable patient positioning^45^ and different sociocultural expectations of the patient populations^45^.

The timing of aesthetic assessment was defined in 48 (64.0 per cent) studies. None of the included studies reported baseline aesthetic data, although four compared preoperative photographs when performing the postoperative assessment^65^^,^^66^^,^^88^^,^^104^. Postoperative assessment most commonly occurred at 6 months (*n* = 11)^2^^,^^36^^,^^39^^,^^40^^,^^65^^,^^112^^,^^119^^,^^122^^,^^126^^,^^131^^,^^136^, then 12 months (*n* = 6)^31^^,^^79^^,^^99^^,^^117^^,^^124^^,^^133^, 5 months^66^, 2 years^128^ or 3 years^47^ (all *n* = 1). Very few (*n* = 2) reported thresholds for assessment, for example at least 6 months^49^ or 2 years^116^ after operation. Other studies used more than one or regular assessments after surgery (*n* = 9)^68^^,^^92–94^^,^^101^^,^^102^^,^^108^^,^^132^^,^^137^ such as every 3–12 months after surgery, or reported a range of time points used for assessment (*n* = 3)^86^^,^^88^^,^^107^. Some investigators assessed aesthetic outcome after adjuvant therapy, at 6 months (*n* = 4)^42^^,^^45^^,^^70^^,^^138^, 12 months (*n* = 1)^134^, regular intervals (*n* = 2)^38^^,^^103^, within a reported range of measurement points (*n* = 2)^90^^,^^106^ or at an unspecified time afterwards (*n* = 4)^58^^,^^85^^,^^120^^,^^129^. Kim and colleagues, measured aesthetic outcome at 6 months after operation or after chemoradiation^125^, if this finished later than 6 months after surgery.

### Quality of life and patient-reported outcome measures

A total of 30 studies (20.3 per cent) reported QOL or other PROMs, in addition to any patient-reported aesthetic outcomes described above (*Table 5*). PROMS which include (but are not limited to) aesthetic outcomes are described in this section. A total of 17 studies used at least one validated outcome measurement tool^30^^,^^43^^,^^47^^,^^54^^,^^56^^,^^57^^,^^61^^,^^67^^,^^85^^,^^94^^,^^100^^,^^102^^,^^104^^,^^105^^,^^117^^,^^124^^,^^139^, mostly frequently the BREAST-Q (*n* = 12)^30^^,^^43^^,^^56^^,^^57^^,^^61^^,^^67^^,^^85^^,^^100^^,^^104^^,^^105^^,^^139^^,^^140^ using three different modules where specified (*Table 6*). Two studies modified the BREAST-Q reduction module to accommodate the TM population by adding items relating to breast cancer treatment and reconstruction^43^^,^^104^.

**Table 5:** Patient-reported and quality-of-life outcome domains

Domain	Outcomes
**Patient satisfaction**	Satisfaction with surgery Satisfaction with the degree to which the reconstructed breast feels a natural part of their body Satisfaction with body Satisfaction with medical team/staff Satisfaction with information provided Satisfaction with breasts when dressed, in underwear/swimwear, when naked* Satisfaction with symmetry* Satisfaction with size* Satisfaction with shape* Satisfaction with cleavage* Satisfaction with how ‘natural’ breast looks* Satisfaction with outcome compared with before surgery* Satisfaction with scar*
**Confidence and self-esteem**	Ability to show oneself in public Self-consciousness Self-confidence Perception of self-image Avoidance of others Social life
**Body image**	Satisfaction with appearance when dressed/in swimwear/naked Difficulty looking at oneself naked Body acceptance Physical attractiveness
**Feelings of normality**	Feeling normal after surgery Feeling ‘feminine’ Feeling the body is ‘whole’ Feeling like other women Feeling equal worth to other women
**Emotional well-being**	Feeling tense/worried/irritable/depressed Difficulty concentrating Poor memory Concern for the future Cognitive functioning Functioning in relationships
**Sexual well-being**	Sexual attractiveness Sexual functioning Sexual confidence clothed and unclothed Comfort in sexual situations Role of breast in sexuality
**Physical well-being**	Breast pain Fatigue Nausea/vomiting Dyspnoea Insomnia Loss of appetite Constipation/diarrhoea Headache Systemic therapy side effects Shock due to hair loss Ability to perform tasks of daily living
**Clothing issues**	Change in clothes worn Comfort with bra
**Recovery time**	Time to get back to work Time to get back to domestic activities/exercise
**Socioeconomic**	Financial difficulties Overall quality of life Choice to have same procedure again

* Items assessing aesthetic outcome which are also included in some patient-reported outcome measures.

**Table 6:** Patient-reported and quality-of-life outcome measures

Outcome measures	Studies*
Validated	
BREAST-Q (all modules)	12 (40.0)
Reduction/mastopexy module	1 (3.3)
BCT module	1 (3.3)
Reconstruction module	3 (10.0)
Modification of existing module/s to suit TM population†	2 (6.7)
Not specified	5 (16.7)
QOL-ACD-B	1 (3.3)
EORTC-QLQ (all)	2 (6.7)
QLQ-C30	2 (6.7)
QLQ-BR23	2 (6.7)
Hopwood Body Image Scale	1 (3.3)
Breast Cancer Treatment Outcomes Scale	2 (6.7)
Non-validated	
Questionnaire/survey‡	8 (26.7)
Verbal questioning or interview	2 (6.7)
Patient chart review	1 (3.3)
Not specified	1 (3.3)

Values in parentheses are percentages.

*Some studies used more than one outcome measure.

†Modification of reduction module to include items relating to reconstruction and breast cancer treatment.

‡ Includes previously published measurement tools which have not been formally validated.BCT, breast-conserving therapy; QOL-ACD-B, quality of life Anti-Cancer Drugs Breast; EORTC-QLQ, European Organisation for Research and Treatment Cancer-Quality of Life Questionnaire. A complete list of patient-reported and quality-of-life outcomes can be found in *Table 5*.

Timing of PROM assessment was specified in 21 (70.0 per cent) studies and varied considerably. Only three studies reported preoperative baseline data^30^^,^^102^^,^^105^. Some assessed PROMs after surgery at 6 months (*n* = 4)^2^^,^^84^^,^^122^^,^^131^, 1 year (*n* = 5)^56^^,^^99^^,^^117^^,^^139^^,^^140^ or 3 years (*n* = 1)^47^. One study (*n* = 1) assessed PROMs a median of 3 months after completion of radiotherapy^57^. Other studies used multiple, regular time points between 3 months and 3 years after surgery (*n* = 7)^30^^,^^41^^,^^43^^,^^74^^,^^92^^,^^94^^,^^102^ or reported a range of time points for measurement (*n* = 3)^85^^,^^104^^,^^124^.

### Functional outcomes

Five (3.4 per cent) studies evaluated functional outcomes, in addition to the PROMs listed above^68^^,^^70^^,^^92^^,^^108^^,^^110^. Four of these reported bilateral mammoreduction techniques^68^^,^^92^^,^^108^^,^^110^. Functional outcomes have been classified into two domains: physical symptoms (pain and arm mobility) and ability to carry out activities of daily living (*Table 7*). None used validated outcome measures and most (*n* = 4) relied on informal verbal questioning^68^^,^^70^^,^^92^^,^^110^. Only one study explicitly stated the timing of assessment (every 3 months after surgery for the first year)^92^.

**Table 7:** Functional outcome domains and outcome measures

Domains	Outcomes	Outcome measures
**Physical symptoms**	Bra strap pain Back pain Shoulder pain Neck pain Mastalgia Vertigo Arm mobility	Use of pain medication or alternative medicine (yoga, chiropractors, massage, physical therapist) Verbal questioning during follow-up Pre- and postoperative Likert scales
**Ability to carry out activities of daily living**	Restriction in physical activities	Number and percentage of cohort

Other functional outcomes contained in quality-of-life and patient-reported outcome measures can be found in *Table 5*.

### Resource use

Twenty-eight studies (18.9 per cent) reported resource use (surrogate measures of cost-effectiveness), in addition to the reoperation and readmission rates described above. The most frequently reported outcomes were total operating time (*n* = 22) and duration of hospital stay (*n* = 18). Two reported total number of interventions per patient^43^^,^^128^ and one reported the total number of postoperative clinic appointments^70^.

## Discussion

This systematic review is the first to summarize comprehensively the outcomes and outcome measures reported in clinical studies of TM, as well as the timing of outcome measurement. With respect to study characteristics, the majority of included articles described small, retrospective cohort studies. Overall, included studies reported outcomes inconsistently across all categories, using mostly non-validated measurement tools, with non-defined or highly variable measurement time points. In particular, aesthetic and QOL outcomes were infrequently reported with few validated PROMs. These findings highlight the need for standardization of reporting through COS development, with a focus on patient and public involvement.

Clinical outcomes relating to oncological safety and surgical morbidity were widely reported, although the majority of reports had a relatively short follow-up period and did not report overall survival, which is considered the gold standard outcome measure for long-term oncological safety^141^. Furthermore, the time interval for measurement of complications was inconsistent and few studies used validated measures of surgical morbidity (such as the Clavien–Dindo classification)^142^ which makes it difficult to compare complication rates reliably across studies and different clinical fields. One-third of studies reported delay (or time) to initiation of adjuvant therapy, but few defined ‘delay’ and those that did used varying thresholds. Time to initiation of adjuvant therapy is significantly associated with adverse outcomes (overall survival, breast-cancer-specific survival and relapse-free survival)^143^^,^^144^. It may be more meaningful, first, to achieve an international consensus definition of what constitutes a clinically important delay to adjuvant therapy^145–147^ and to measure the percentage of the cohort that meets this standard. A minority of studies reported practicalities such as duration of drain insertion or investigation of irregular breast symptoms after operation. National surveys have demonstrated wide variation regarding use of drains in oncoplastic breast surgery^148^ and practical issues relating to surgery are important to patients^149^.

Few relevant studies reported rates of contralateral breast cancer or the histological examination of excised tissue for this reason. Whilst it is rare to find imaging occult contralateral disease in sporadic breast cancers following TM^150^, it may be important to monitor this as the practice of TM increases to support clinical and patient decision-making.

BCS^12^ and TM^15^ are demonstrably oncologically safe, although long-term data for the latter are limited^151^. The decision to proceed with TM is therefore likely to be driven by aesthetic and QOL considerations, which should have a strong evidence base. However, only half of included studies examined aesthetic outcomes, mostly using non-validated scoring systems or questionnaires. Studies which did use validated outcome measures for BCS used a variety of scoring systems (Harris scale^90^^,^^92^^,^^98^^,^^111–113^, Garbay criteria^45^, Regnault and Bostwick classification^90^), all of which were first described in the 1970s–1990s^152–154^. Fewer studies used BCCT.core software, probably because it was not described until 2012. In one-third of studies that reported aesthetic outcome, it was evaluated only by clinicians without any patient input. A significant minority of studies also used non-medical observers in aesthetic rating panels, however justification for their role is unclear. Ultimately, it will be important to engage patients to ask how they believe aesthetic outcome should be assessed, and by whom, particularly as the few studies that assessed correlation found disagreements between patients’ and clinicians’ ratings^45^^,^^85^.

One-fifth of studies examined QOL, most commonly using the BREAST-Q. This finding should be interpreted in the context of a non-date-restricted search, in that high-quality PROMs have been developed fairly recently (the BREAST-Q was not created until 2009)^155^. At least three different modules (reduction/mastopexy, BCT, reconstruction) were utilized, where specified. In two studies, authors also modified the BREAST-Q reduction module by adding items relating to breast cancer treatment and reconstruction, which is not permitted by the BREAST-Q user manual^156^. This suggests that the applicability of BREAST-Q modules for TM patients should be reviewed and possibly adapted, taking account of the different types of mammaplasty performed.

With regard to both aesthetic and QOL outcomes, many studies failed to define the timing of outcome measurements. Where temporal data capture was defined, it varied substantially with different benchmarks, such as after surgery or after adjuvant therapy. Future studies should report timing of outcome measurement, since aesthetic outcomes are dynamic and may change over time and following adjuvant radiotherapy^157^. Very few studies reported baseline aesthetic and QOL data, despite the fact that preoperative concerns regarding appearance (for example, macromastia or ptosis) may partially motivate patient treatment decisions for TM.

Alleviation of functional symptoms associated with macromastia is a cited indication for TM^4^, but a minority of studies explored this outcome, either within a validated PROM or using non-validated author-generated measures. Furthermore, few specified the timing of functional assessment. This is particularly important because time since surgery and adjuvant radiotherapy are known confounders of functional outcomes after breast surgery^158^.

Resource use was reported inconsistently. Increasing use of TM presents a new paradigm in breast surgery, whereby more than one oncological procedure (TM, traditional BCS and/or mastectomy) may be safe for certain patients. Consideration of cost-effectiveness, in addition to patient choice, may help to inform care pathways particularly in publicly funded healthcare systems.

The strengths of this systematic review include its unique and comprehensive evaluation of the state of outcome reporting in TM, using four electronic databases searched from inception. However, the findings are subject to some limitations. The search was language-restricted and may have missed otherwise eligible non-English articles. The search was not date-restricted and hence the review probably underestimates the proportion of current studies undertaking high-quality PROM assessment. The aim of the review was to evaluate outcome reporting comprehensively in TM; as a result, a heterogeneous group of studies of variable quality and reporting was included, which may not represent recent, larger and higher-quality studies. Formal evaluation of outcome measure validity according to COSMIN methodology^159^ was considered outside the scope of this review, but is planned.

There is a lack of standardization in outcome reporting for TM. This inhibits high-quality evidence synthesis used to inform best medical practice. Development of a COS will strengthen particularly the evidence base for aesthetic, QOL and functional outcomes of TM, thereby facilitating informed patient selection and increased uptake in oncoplastic breast units. The limited use of PROMs to date highlights the importance of patient and public involvement in this process. The available outcome measures have been summarized with a view to assessing formally their validity and technological applications for aesthetic and functional assessment. The field will also benefit from more high-quality, prospectively designed studies with larger participant numbers, which can be achieved through research collaboratives such as the TeaM Study^14^^,^^137^.

## Supplementary material

Supplementary material is available at *BJS Open* online.

## Funding

This work is independent research funded by the National Institute for Health Research (NIHR) Imperial Biomedical Research Centre (BRC). The views expressed in this publication are those of the authors and not necessarily those of the NHS, the NIHR or the Department of Health.

*Disclosure*. The authors declare no conflicts of interest.

## Supplementary Material

zrab126_Supplementary_Data
