# Supplementary material for: Outcome reporting in therapeutic mammaplasty: a systematic review
Source: BJS Open. 2021 Dec 11;5(6):zrab126. doi: 10.1093/bjsopen/zrab126 (PMC8665419; doi:10.1093/bjsopen/zrab126)
Supplement: zrab126_Supplementary_Data [file zrab126_supplementary_data.docx]

**Table S1.** Inclusion and exclusion criteria.

| **Inclusion criteria** | **Exclusion criteria** |
| --- | --- |
| - Randomised and non-randomised trials, cohort studies and case-control studies - Adult female participants undergoing TM as primary treatment for breast cancer (including both immediate and delayed symmetrisation) - TM techniques (level 1-2 oncoplastic breast surgery) including the following skin incision patterns: Wise, vertical scar, peri- or circumareolar, Grisotti, melon slice (horizontal wedge excision) | - Study design: systematic reviews, meta-analyses, case series, case reports, conference abstracts, and animal, cadaveric or laboratory studies - Non-English language articles - Non-oncological breast surgery - Studies which do not report TM techniques (total mastectomy ± reconstruction ± symmetrisation; standard BCS) - Studies with male participants or those who are < 16 years old - Articles which do not report patient outcomes - BCS combined with volume replacement procedures including but not limited to implants, latissimus dorsi mini-flaps, thoracodorsal artery perforator flaps, lateral intercostal artery perforator flaps |

Abbreviation: BCS, breast conserving surgery; TM, therapeutic mammaplasty

**Table S2.** Example search strategy for Ovid Medline.

| **Search concept** | | |
| --- | --- | --- |
| Therapeutic mammaplasty | Breast cancer | Study design |
| 1. (therapeutic adj3 mamm?plast*).mp. 2. reduction mamm?plast*.mp. 3. oncoplastic breast surger*.mp. 4. Mammaplasty/ 5. 1 or 2 or 3 or 4 | 1. exp Breast Neoplasms/ 2. breast neoplasm*.mp. 3. (breast adj2 cancer*).mp. 4. (breast adj2 tumo?r*).mp. 5. 6 or 7 or 8 or 9 | 12. randomized controlled trial.pt.  13. controlled clinical trial.pt.  14. randomi?ed.ab.  15. placebo.ab.  16. drug therapy.fs.  17. randomly.ab.  18. trial.ab.  19. groups.ab.  20. 12 or 13 or 14 or 15 or 16 or 17 or 18 or 19  21. exp cohort studies/  22. cohort$.tw.  23. controlled clinical trial.pt.  24. epidemiologic methods/  25. limit 24 to yr="1966 - 1989"  26. exp case-control studies/  27. (case$ and control$).tw.  28. 21 or 22 or 23  29. 24 or 25 or 26 or 27  30. 28 or 29 |
| 1. Combined search for therapeutic mammaplasty AND breast cancer = (5 AND 10) | | 31. Trial OR cohort study OR case-control study= (20 OR 30) |
| Overall search = (11 AND 31) | | |

**Table S3.** List of included studies

| **First Author** | **Year published** | **Country** | **Study period** | **Study type** | **Prospective/ retrospective** | **Type of therapeutic mammaplasty** | **Skin incision pattern** | **Contralateral surgery included?** | **Radiotherapy included?** |
| --- | --- | --- | --- | --- | --- | --- | --- | --- | --- |
| De Lorenzi | 2016 | Italy | 2000-2008 | Matched cohort analysis | Retrospective | Glandular reconstructions incl all local and regional flaps, therapeutic mammaplasties | Multiple | Y | N |
| Acea-Nebril | 2017 | Spain | 2000-2006 | Cohort | Retrospective | Vertical wise pattern | Wise pattern | Y | Y |
| Bamford | 2015 | UK | Over 8 years | Cohort | Retrospective | Wise pattern technique; single vertical scar incision | Wise pattern | Y | Y |
| Adimulam | 2014 | India | 2007-2009 | Cohort | Prospective | Oncoplastic technique varied depending on the location of tumor, size of the breast and volume of the breast tissue excised | Multiple | N | Y |
| Aljarrah | 2011 | France | August 2005 to March 2009 | Cohort | Prospective | Oncoplastic "crescent" technique | Other | N | Y |
| Chang 2012 | 2012 | US | 2000-2009 | Cohort | Retrospective | simultaneous partial mastectomy/reduction mammoplasty procedures | Wise pattern | Y | Y |
| Chen | 2014 | Taiwan | June - December 2011 | Cohort | Prospective | Modified round block mammaplasty | Peri-areolar/circumareolar with skin excision (round block, Benelli, racquet) | ? | ? |
| Crown | 2018 | US | December 2012 - July 2015 | Cohort | Retrospective | Oncoplastic reduction mammaplasty | Wise pattern | Y | Y |
| Currie | 2013 | UK | 2007-2011 | Cohort | Retrospective | Reduction mammaplasty | Wise pattern | Y | Y |
| Eaton | 2014 | US | January 1994 - December 2010 | Cohort | Retrospective | Oncoplastic reduction mammaplasty | Multiple | Y | Y |
| Smith | 1998 | US | 1988-1996 | Case series | Retrospective | Bilateral reduction mammaplasty | Wise pattern | Y | Y |
| Spear | 2003 | US | not stated | Case series | Retrospective | Partial mastectomy and reduction mammaplasty principles | Unclear | Y | Y |
| Stein | 2020 | Canada | 2014-2018 | Cohort | Retrospective | Therapeutic reduction mammaplasty | Unclear | Y | Y |
| Szynglarewicz | 2016 | Poland | 2008-2014 | Cohort | Prospective | Breast segmentectomy with rotation mammaplasty | Other | N | Y |
| Yamashita | 2006 | Japan | December 2001-March 2006 | cohort | Retrospective | extended wide resection with mobilisation of mammary gland, transplantation of lateral tissue flap, filling with absorbent synthetic fiber mesh or cotton | Other | N | Y |
| Yang | 2011 | South Korea | January 2006-August 2009 | Cohort | Retrospective | Round block, batwing mastopexy, tennis racket, rotational flap, parallelogram mastopexy lumpectomy | Multiple | Y | Y |
| Youssef | 2018 | Egypt | 2013-2015 | Cohort feasibility study | Prospective | Level I, II and III oncoplastic breast surgery | Multiple | Y | Y |
| Ho | 2016 | UK | November 2010 - October 2015 | cohort | Retrospective | matrix rotation | Unclear | Y | Y |
| Kelemen | 2019 | Budapest | January 2010- January 2016 | cohort | retrospective | therapeutic mammaplasty, dermoglandular rotation, periareolar | Multiple | N | Y |
| Tong | 2016 | US | January 2005-April 2013 | cohort | Retrospective | oncoplastic breast repair following partial mastectomy | Multiple | Y | Y |
| Lee | 2015 | Korea | January 2007-June 2013 | Cohort | Prospective | BCS, repositioning of adjacent breast tissue and insertion of Interceed | Other | N | Y |
| Schrenk | 2006 | Austria | January 1997- December 2004 | Cohort | Prospective | tumour quadrantectomy with therapeutic mammaplasty | Multiple | Y | Y |
| van la Parra | 2019 | France | January 2004-February 2018 | Cohort | Retrospective | level II mammaplasties | Multiple | Y | Y |
| DeBiasio | 2016 | Italy | January 2012-January 2014 | Cohort | Retrospective | quadrantectomy of the lower breast pole and volume replacement with remod- elling through three posterior scorings | Other | N | Y |
| Colombo | 2015 | France | 2011-2014 | Cohort | Retrospective | modified McKissock mammaplasty | Wise pattern | Y | Y |
| Goffman | 2005 | US | over 7 years | cohort | retrospective | bilateral mammoreduction | Wise pattern | Y | Y |
| Harvey | 2014 | UK | 2009-2013 | cohort | retrospective | inferior/superior pedicle, grisotti flap, wedge technique, wise reduction, vertical scar, periareolar, comma | Multiple | Y | Y |
| Gulcelik | 2011 | Turkey | not stated | cohort | retrospective | reduction mammaplasty | Unclear | Y | Y |
| Dogan | 2012 | Turkey | June 2010-June 2011 | cohort | prospective | intraglandular flap technique using a racquet incision | Peri-areolar/circumareolar with skin excision (round block, Benelli, racquet) | N | Y |
| van Paridon | 2017 | US | 2010-2015 | cohort | retrospective | complex layered closure, local tissue rearrangement, pedicled flap, mastopexy, bilateral reduction, or implant placement | Multiple | Y | Y |
| Carstensen | 2017 | Denmark | 2011-2014 | cohort | unclear | BCS and over Wise mammoplasty | Wise pattern | Y | Y |
| Caruso | 2008 | Italy | November 1994-December 2001 | cohort | retrospective | bilateral breast reduction | Multiple | Y | Y |
| Abdelhamid | 2018 | Egypt | October 2012- September 2014 | Cohort | Prospective | Lazy lateral technique | Other | N | Y |
| Fitzal | 2007 | Austria | September 2005- September 2006 | case series | not stated | Hall-Findlay technique (breast reduction) | Single vertical scar (Le Jour) | Y | Y |
| Emiroglu | 2017 | Turkey | 1996-2011 | cohort | retrospective | oncoplastic reduction mammaplasty | Multiple | Y | Y |
| Lim | 2017 | Singapore | 1st May 2014 - 31st January 2016 | matched case control study | retrospective | oncoplastic round block technique | Peri-areolar/circumareolar with skin excision (round block, Benelli, racquet) | N | Y |
| Santos | 2015 | Brazil | 2007-2012 | cross-sectional, multicentric, two-independent-group study | prospective | bilateral surgeries with mammaplasty techniques | Multiple | Y | Y |
| Chauhan | 2016 | India | January 2012-August 2014 | cohort | prospective | volume displacement (periareolar, superior and inferior pedicle techniques, quadrantectomy with glan- dular remodeling, and dermo-glandular flaps) or volume replacement (mini LD myofascial or myocutneous flap) | Multiple | Y | Y |
| DiMicco | 2017 | UK | June 2009-November 2014 | cohort | retrospective | bilateral mammaplasty (wise, round block, short scar periareolar inferior pedicle, lateral, omega, vertical) | Multiple | Y | Y |
| Kelemen | 2019 | Budapest | February 2011-January 2017 | cohort | retrospective | therapeutic modified Wise‐pattern OBCS with immediate or delayed contralateral symmetrization | Wise pattern | Y | Y |
| Rezai | 2015 | Germany | 2004-2009 | case cohort trial | Retrospective | glandular rotation mammaplasty, reduction mammaplasty, fat tissue displacement, thoracoepigastric flap/lateral thoracic advancement flap | Multiple | Y | Y |
| Han | 2010 | Germany | January 2005-October 2006 | cohort | retrospective | oncological breast reduction for hypermastia* | Unclear | Y | Y |
| Manie | 2020 | Egypt | May 2016 - June 2018 | cohort | prospective | batwing mammoplasty and contralateral symmetrisation | Other | Y | Y |
| Losken | 2010 | US | 2001-2007 | Cohort | Retrospective | Oncoplastic reduction mammaplasty | Unclear | Y | Y |
| Munhoz | 2007 | Brazil | 1999-2006 | Cohort | Retrospective | Oncoplastic reduction mammaplasty (inferior pedicle) | Other | Y | Y |
| Matrai | 2019 | Hungary | 2016-2017 | Cohort | Retrospective analysis of a prospective database | Level 1 retroglandular oncoplastic technique (via IMF) | Other | N | Y |
| Munhoz 2011 | 2011 | Brazil | 1999-2009 | Cohort | Retrospective | Oncoplastic reduction mammaplasty | Multiple | Y | Y |
| Lin | 2016 | Taiwan | 2012-2015 | Cohort | Retrospective | Oncoplastic mammaplasty (matrix rotation technique) | Multiple | N | Y |
| Losken | 2014 | US | 2009-2013 | Cohort | Retrospective | Not stated | Unclear | Not stated | Y |
| Kaviani | 2020 | Iran | 2008-2018 | Cohort | Retrospective | Oncoplastic reduction mammoplasty (variety of techniques) | Multiple | Y | Y |
| Kim | 2012 | Korea | Not stated | Cohort | Prospective | Oncoplastic redcuction mammoplasty (lateral breast rotation flap) | Other | Not stated | Y |
| Koppiker | 2019 | India | Not stated | Cohort | Retrospective | Oncoplastic reduction mammaplasty (Wise pattern or vertical skin incision) | Multiple | Y | Y |
| Lim | 2016 | Singapore | 2014-2015 | Cohort | Retrospective | Variable oncoplastic mammoplasty (most commonly round block) | Multiple | Not stated | Y |
| Mathapati | 2019 | India | 2015-2016 | Cohort | Propsective | Oncoplastic reduction mammoplasty (various techniques) | Multiple | N | Y |
| Mattingly | 2017 | US | 2008-2014 | Cohort | Retrospective | Oncoplastic reduction mammoplasty | Unclear | Y | Y |
| Mazouni | 2013 | France | 2002-2010 | Cohort | Retrospective | Oncoplastic reduction mammoplasty | Multiple | N | Y |
| McCullley | 2005 | UK | Not stated | Cohort | Retrospective | Oncoplastic reduction mammoplasty (skin incision either wise pattern or vertical; variable pedicles) | Multiple | Y | Y |
| Chauhan | 2016 | India | January 2012 - December 2014 | Comparative cohort (Oncoplastic vs Breast conservation) | Prospective | Oncoplastic procedures* | Multiple | N - Offered after adjuvant therapy completed | Yes |
| Christiansen | 2008 | US | 2001-2005 | Cohort | Prospective | Reduction mammaplasty with omega incision | Other | Y | Y |
| Cil | 2016 | Canada | 2005-2014 | Comparative cohort (Lumpectomy vs Oncoplastic) | Retrospective | Oncoplastic mammoplasty with soft tissue transfer | Multiple | Contralateral mastectomy patients were excluded | ? |
| Clough | 2012 | France | 2005-2010 | Cohort | Prospective | Quandrant - specific oncoplastic technique | Multiple | Y | Y |
| Clough | 2003 | France | July 1985 - June 1999 | Cohort | Prospective | Oncoplastic mammoplasty | Multiple | Y | Y |
| Clough | 1995 | France | May 1986 - February 1993 | Cohort | Prospective | Wide lumpectomy w/ remodelling mammoplasty | Multiple | Y | Y |
| Clough | 2013 | France | 2004-2011 | Cohort | Retrospective | Lower inner quadrant-V mammoplasty (LIQ-V) | Other | Y | Y |
| Crown | 2019 | US | January 2017- June 2018 | Cohort | Prospective | Oncoplastic central partial mastectomy and neoareolar reduction mammoplasty w/ immediate nipple reconstruction | Wise pattern | Y | Y |
| Deigni | 2020 | US | January 2010- January 2016 | Comparative cohort (immediate vs delayed contralateral surgery) | Retrospective | Oncoplastic mastopexy/breast reduction and contralateral symmetrising mastopexy | Multiple | Y | Y |
| Denewer | 2012 | Egypt | ? | Cohort | Prospective | Reduction mammaplasty using superior and superomedial pedicles | Other | Y | Y |
| DiMicco | 2017 | UK | June 2009 - November 2014 | Comparative cohort (BCS vs Bilateral reduction mammoplasty) | Retrospective | Reduction mammaplasty | Multiple | Y | Y |
| Eichler | 2013 | Germany | 2007 | Comparative cohort (Lumpectomy vs Matopexy) | Retrospective | Mastopexy | Multiple | ? | Y |
| Emiroglu | 2016 | Turkey | January 1996 - May 2011 | Cohort | Retrospective | Oncoplastic reduction mammaplasty; wise pattern, vertical incision | Multiple | Y | Y |
| Farouk | 2015 | Egypt | June 2011 - December 2014 | Cohort | Prospective | Oncoplastic techniques (dependent on free safety margins, breast volume and ptotic degree) | Multiple | Y (no patients opted for contralateral surgery) | Y |
| Gulcelik | 2013 | Turkey | 2003-2010 | Comparative cohort (Breast conserving surgery vs Bilateral reduction mammoplasty) | Prospective | Reduction mammaplasty | Multiple | Y | Y |
| Hashem | 2017 | Egypt | 2009-2015 | Comparative cohort | Retrospective | Batwing mammaplasty vs Wise pattern | Multiple | ? | ? |
| Emiroglu | 2015 | Turkey | March 2000- October 2011 | Cohort | Retrospective | Oncoplastic procedures* | Multiple | Y | Y |
| Santanelli | 2009 | Italy | February 2005-November 2007 | Cohort | Prospective | Reduction mammoplasty: Quadrantectomy (modified Wise-pattern) | Wise pattern | Y | Y |
| Nisiri | 2018 | Iran | 2010-2013 | Comparative cohort | Prospective | Oncoplastic mammoplasty | Multiple | ? | Y |
| Mansell | 2017 | UK | June 2009- August 2012 | Comparative cohort | Prospective | Oncoplastic mammoplasty | Multiple | Y | Y |
| Rietjens | 2007 | Italy | September 1994-December 1999 | Cohort | Prospective? | Breast conserving therapy | Multiple | Y | Y |
| Tenofsky | 2014 | US | December 2006 - April 2011 | Comparative cohort | Retrospective | Oncoplastic lumpectomy | Multiple | Y | Y |
| Ng | 2016 | Australia | July 2012 - July 2013 | Cohort | Retrospective | Oncoplastic 'Crescent' technique | Other | N | Y |
| Roughton | 2012 | US | March 2003 - September 2009 | Cohort | Retrospective | Oncoplastic mammoplasty | Multiple | Y | Y |
| De Lorenzi | 2018 | Italy | 2000-2008 | Case-control | Retrospective | Oncoplastic surgery | Unclear | N? | Y |
| Fosh | 2014 | Australia | 1995-2009 | Cohort | Retrospective | Oncoplastic techniques | Unclear | ? | ? |
| Kabir | 2016 | UK | August 2005-September 2010 | Cohort | Retrospective | Oncoplastic reduction mammaplasty | Multiple | Y | Y |
| Imahiyerobo | 2015 |  | March 2005- March 2012 | Comparative cohort | Retrospective | Oncoplastic reduction mammaplasty | Multiple | N | Y |
| Kijima | 2011 | Japan | January 2007 - May 2009 | Cohort | Prospective | Oncoplastic reduction mammoplasty for lateral quadrant breast cancer | Other | Y | Y |
| Song | 2010 | US | January 1991-June 2006 | Cohort | Retrospective | Lumpectomy and reduction mammoplasty | Multiple | Y | Y |
| Schaverien | 2013 | UK | not stated | Cohort | Retrospective | Oncoplastic techniques | Multiple | Y | Y |
| Acosta-Marin | 2014 | Venezuela | January 2011-October 2012 | Cohort | Prospective | Level II 'oncoplastic surgery ' versus 'standard' BCS | Multiple | N | N |
| Agrawal | 2018 | India | January 2009 to June 2014 | Cohort | Retrospective | Volume displacement techniques (rotation and advancement of fibro-glandular flaps of the breast) and mammoplasty (levelII), reconstruction inclduing local and regional flaps | Multiple | N | Y |
| Amitai | 2018 | Israel | 2009-2014 | Cohort | Prospective | Immediate partial breast reconstruction using local tissue rearrangement with breast reduction and/or mastopexy pattern techniques or breast augmentation pattern technique | Multiple | Y | Y |
| Angarita | 2020 | Canada | 2005 -2016 | Cohort | Retrospective | Traditional BCS, 'Adjacent tissue transfer of the trunk', mastopexy or reduction mammoplasty | Multiple | N | N |
| Bali | 2018 | UK | April 2014-September 2016 | Cohort | Retrospective | WLE or OBS (parenchymal displacement surgery such as therapeutic mammoplasty or mastopexy) or parencyhmal replacement surgery (such as partial breast reconstruction with chest wall perforator flap) | Multiple | N | N |
| Barnea | 2014 | Israel | Setember 2006 - June 2010 | Cohort | Prospective | Vertical scar superior-medial pedicle reduction pattern | Single vertical scar (Le Jour) | Y | Y |
| Behluli | 2019 | Germany | January 2012-October 2014 | Cohort | Retrospective | Conventional BCS versus Oncoplastic Breast Surgery (level II techniques including therapeutic mammoplasty, vertical mammoplasty (medial or supero-medial pedicle), inverted T pattern therapeutic mammoplasty, V-mammoplasty, racquet technique and round block mammoplasty) | Multiple | N | N |
| Bogusevicius | 2014 | Lithuania | 2003-2005 | Cohort | Prospective |  | Multiple | N | Y |
| Bong | 2010 | USA | March 2004-December 2009 | Cohort | Retrospective | Oncoplastic mastopexy reconstruction (Batwing, Parallelogram, Modified Wise, Miscellaneous) | Multiple | N | Y |
| Bordoni | 2019 | Italy | March 2014-March 2016 | Cohort | Not stated | Upper-inner quadrant therapeutic mammoplasty | Other | Y | Y |
| Burrah | 2020 | UK | December 2011-December 2017 | Cohort | Prospective | Upper-inner quadrant therapeutic mammoplasty | Peri-areolar/circumareolar with skin excision (round block, Benelli, racquet) | Y | N |
| Calabrese | 2018 | italy | January 2000 - December 2010 | Cohort | Prospective | Donut mastopexy, Superior pedicle wise pattern, inferior pedicle wise pattern, grisotti, comma shape, medial pedicle mastopexy, lateral pedicle mastopexy | Multiple | N | Y |
| CaliCassi | 2016 | Italy | January 2012-December 2014 | Cohort | Retrospective | Therapeutic mammoplasty and adjacent tissue transfer following lumpectomy | Unclear | N | Y |
| Carter | 2016 | USA | January 2007- December 2014 | Cohort | Prospective | Breast conserving surgery, BCS with reconstruction, total mastectomy or therapeutic mammoplasty with immediate reconstruction | Unclear | N | Y |
| Chakravorty | 2012 | UK | June 2003 - Feb 2010 | Cohort | Retrospective | Standard BCS versus Oncoplastic BCS (Wise pattern, coma and lateral, Grisotti, and Benelli (round block) | Multiple | N | Y |
| Chang | 2004 | USA | 1998-2003 | Cohort | Retrospective | Wise pattern technique | Wise pattern | Y |  |
| Down | 2013 | UK | July 2006- April 2010 | Cohort | Not stated | Therapeutic mammoplasty (not stated), sub-axillary fat pad rotation mammoplasties, thoraco-epigastricflaps and 1 central flap | Multiple | Y | Y |
| Clough | 2018 | France | January 2014-March 2016 | Cohort | Prospective | Lateral mammoplasty, J mammoplasty, V mammoplasty, super pedicle mammoplasty, inferior pedicle mammoplasty | Multiple | Y | Y |
| JeeyeonLee | 2017 | Korea | 2008-2013 | Cohort | Prospective | Glandular flap, V-Y advanced flap, purse, string suture, adipofacial turnover flap, round block technique, batwing mastopexy, tennis racket technique, rotating flap, reduction mammoplasty | Multiple | N | Y |
| Losken | 2002 | USA | January 1991-December 2000 | Cohort | Not stated | Wise pattern design | Wise pattern | Y | Y |
| Nizet | 2015 | Belgium | September 2006 - August 2013 | Cohort | Retrospective | Tumour resection with unilateral oncoplastic surgery (superior pedicle, superomedial pedicle, superolateral pedicle, inferior pedicle, Grisotti flaps, Thorek's technique) with or without simultaneous contralateral breast reduction | Multiple | Y | Y |
| Losken | 2007 | USA | 1991 and April 2006 | Cohort | Retrospective | Wise and vertical | Multiple | Y | N |
| Egro | 2015 | USA | January 1995 - December 2012 | Cohort | Prospective | Reduction mammaplasty | Unclear | Y | N |
| Borm | 2019 | Germany | January 2000-December 2005 | Cohort | Retrospective | Breast conserving surgery (not stated) | Multiple | Y | Y |
| Bordoni | 2018 | Italy | March 2014-March 2016 | Cohort | Not stated | Vertical or inverted T | Multiple | Y | Y |
| Sanchez | 2020 | Italy | January 1998 - January 2018 | Cohort | Prospective | Inverted T, J mammaplasty, Round block, Grisotti, Batwing | Multiple | Y | Y |
| Romics | 2018 | UK | September 2005 - March 2017 | Cohort | Retrospective | Level II oncoplastic breast surgery as defined by Clough et al (to include wise pattern reduction, round block, LICAP/TDAP/LTAP, regnault B-plasty, Grisotti flap, vertical lejour mammoplasty, matrix rotation/J mammoplasty, Thoraco-epigastric flap, Lateral/medial mammoplasty, melon slice reduction, Crescent flap, Batwing mammoplasty, VY lateral advancement, V - mammoplasty, skin pouch mammoplasty, S-mammoplasty, rotational advancement flap, local flap (other), unknown | Multiple | Y | Y |
| Meretoja | 2010 | Finland | January 2005-December 2007 | Cohort | Prospective | Areola transposition, fibroglandular advancement only, reduction mammaplasty, central resection, planned mastectomy, converted mastectomy | Unclear | Y | Y |
| Huemer | 2007 | Austria | September 1998-January 2005 | Cohort | Not stated | Inferior based pedicle type closure, Benelli Grisotti, Direct closure, Inverse T Closure | Multiple | Y | Y |
| Grubnik | 2013 | South Africa | 2002-2009 | Cohort | Retrospective | Wise or modified with extended pedicle with or without skin diskc, canted wise, hemibatwing | Multiple | Y | Y |
| Munhoz | 2006 | Brazil | January 1999 - October 2005 | Cohort | Retrospective | Oncoplastic reduction mammaplasty: modified Wise pattern | Wise pattern | Y | Y |
| Munhoz | 2006 | Brazil | January 1999 - August 2004 | Cohort | Retrospective | Oncoplastic reduction mammaplasty | Wise pattern | Y | Y |
| O'Connell | 2018 | UK | September 2016 - June 2017 | Cohort | Prospective | the application of breast reduction or mastopexy techniques, including removal of skin to reduce the skin envelope, to treat invasive or ductal carcinoma in situ (DCIS) using BCS | Multiple | (1/3 Y) | 92.2 Y to either chemo or radio |
| Potter | 2020 | UK | July 2016 - December 2016 AND September 2016 - June 2017 (Paper combined subsets from the TeaM and iBRA-2 studies) | Subset analysis of 2 cohorts | Prospective | Several techniques: "breast reduction or mastopexy techniques" | Multiple | Y | Y |
| Pearce | 2020 | USA | 1993 - 2016 | Cohort | Prospective | unspecified level 2 oncoplastic surgery | Unclear | Y | Y |
| Qureshi | 2014 | Pakistan | December 2009 - November 2011 | cohort | Prospective | Superior pedicle mammoplasty technique with an inverted T-scar was done for tumours situated in the central or inferior quadrants of the breast; lateral mammoplasty technique was used. It included the fusiform and j mammoplasty techniques | Multiple | N | Y |
| Resende Paulinelli | 2020 | Brazil | March 2007 and October 2019 | Cohort | Retrospective | geometric compensation mammaplasty | Other | Y | Y |
| Rezai | 2015 | Germany | 2004 - 2009 | Cohort | Retrospective | For all locations of the upper hemisphere of the breast and unicentric tumours, glandular rotation mammaplasty was the standard option for reshaping of the breast. With multicentricity or breast resection[20 % or tumours of the lower hemisphere of the breast, a reduction mammaplasty pattern was applied (inferior-pedicled technique described by Ribeiro in the modification of the author) to reconstitute the optimal breast form. This procedure avoids birds peak deformations for patients with gross resection of tissue in the lower quadrants of the breast. Where fat tissue was readily accessible for volume displacement without necessity of musculocutaneous flaps, this was incorporated in the concept of reshaping of the breast such as the thoracoepigastric flap for the lower quadrants (in cases of skin resection) and lateral thoracic advancement flap for the upper outer quadrant (in cases with need of additional volume replacement). | Multiple | Y | unspecified |
| Rose | 2014 | Denmark | January 2008 - December 2010 | Cohort | Prospective | The volume reduction technique involves tumour resection along with the normal tissue resected in a reduction mammoplasty. Therefore, the partial mastectomy is integrated into the reduction mammoplasty procedure. Conversely, in the volume displacement technique, the defect after partial mastectomy is filled with internal flaps of breast tissue, whereas the replacement technique involves filling the defect with external flaps of tissue from outside the breast on the thoracic wall. When the reconstruction was done using the volume reduction or volume displacement technique, reduction mammoplasty or mastopexy was simultaneously performed on the contralateral breast to ensure symmetry. | Unclear | Y | Y |
| Roshdy | 2015 | Egypt | June 2011 - December 2014 | Cohort | Prospective | LeJour pattern modified according to the tumor location | Single vertical scar (Le Jour) | N (all patients refused) | Y |
| Shekhawat | 2015 | India | January 2013 - October 2014 | Cohort | Prospective | Oncoplastic reduction mammaplasty: unknown technique | Unclear | Y | Y |
| Shin | 2018 | Korea | January 2013 - December 2016 | Cohort | Retrospective | Glandular tissue reshaping, Reduction oncoplasty, LD flap transposition (endoscopic-assisted LD flap transposition) | Multiple | Y | Y |
| Silverstein | 2015 | USA | - | Cohort | prospective | standard wise pattern reduction or split reduction procedure | Wise pattern | Y | Y |
| Caruso | 2011 | Italy | - | Cohort | Retrospective | Oncoplastic reduction mammaplasty: inferior, superior and supero-medial pedicle breast reductions, Grisotti’s flap for central quadrantectomies, comma shaped mammaplasties and Benelli’s mastopexies | Multiple | - | Y |
| Akyurek | 2019 | USA | January 2011 - January 2017 | Cadaver + Cohort | Prospective | oncoplastic reduction mammaplasty: Vertical mastopexy | Single vertical scar (Le Jour) | Y | Y |
| Patel | 2011 | USA | 2003 - 2009 | Cohort | Retrospective | oncoplastic reduction mammaplasty | Unclear | - | Y |
| VallejodaSilva | 2007 | Brazil | 2003 - 2006 | Cohort | Retrospective | oncoplastic reduction mammaplasty: Liacyr I Liacyr III Liacyr V Pitanguy Latissimus dorsi L-shape Round block Atypical | Multiple | Y | Y |
| Nos | 1998 | France | May 1986- August 1996 | Cohort | Retrospective | Oncoplastic reduction mammaplasty: inverted T scar, inverted V scar, or a vertical scar | Multiple | Y | Y |
| Singh | 2018 | India | January 2013 - April 2016 | Cohort | Retrospective | Oncoplastic reduction mammaplasty: Lateral Oncoplastic Breast Surgery | Other | - | Y |
| Semprini | 2013 | Italy | November 2005 - December 2010 | Cohort | prospective | quadrantectomy and post-quandrantectomy breast reshaping. NOTE that inferior quadrantectomies were followed with advanced techniques, specifically mentioning a "key hole" reduction mammaplasty | Multiple | Y | Y |
| Rose | 2019 | Denmark | 2008 - 2013 | Cohort | Prospective | OBS included a not specified TM technique | Unclear | Y | Y |
| Shechter | 2019 | Israel | January 2011 - December 2016 | Cohort | Retrospective | reduction mamaplasty: initially Wise pattern but afterwards round block? | Multiple | Y | Y |
| Kronowitz | 2007 | USA | 1990 - 2002 | Cohort | Retrospective | partial mastectomy: parenchymal pedicle of deepithelialized breast tissue with or without an intact nipple-areola complex. | Multiple | Y | Y |
| Ojala | 2017 | Finland | 2010 | Cohort | Retrospective | racket mammoplasty 19 (22%), reduction mammoplasty techniques 19 (22%), round block 16 (19%), rotationplasty techniques 16 (19%), extensive dual plane undermining 12 (14%) and other oncoplastic techniques 4 (5%). | Multiple | Y | Y |
| Kaviani | 2014 | Iran | 2007 to 2012 | Cohort | Prospective | oncoplastic reduction: several techniques including round block, tennis racket, inverted T, V type | Multiple | Y | Y |
| Kelsall | 2017 | UK | 1999 - 2014 | Cohort | Prospective | oncoplastic reduction | Unclear | Y | Y |
| Lee | 2018 | Korea | 2008 - 2013 | Cohort | Retrospective | Batwing mastopexy, Glandular reshaping, Round block technique, Purse-string suture technique, Tennis racket technique, Local flap, Rotating flap, Reduction mammoplasty | Multiple | - | Y |
| Wijgman | 2017 | Netherlands | January 2010 - December 2014 | Cohort | Retrospective | oncoplastic mammaplasty technique | Multiple | - | Y |
| Peled | 2014 | USA | 2001 and 2010 | Cohort | Retrospective | oncoplastic mammaplasty technique | Wise pattern | Y | Y |
| Knowles | 2020 | Canada | 2009-2015 | cohort | Retrospective | oncoplastic breast surgery unspecified | Unclear | Y | Y |
|  |  |  |  |  |  |  |  |  |  |

**Table S4.** Long term oncological safety outcomes

| **Oncological safety outcome** | **Studies, N (%)^a^** | **Range of time points for measurement** |
| --- | --- | --- |
| Incidence or rate of local recurrence | 100 (68) | From 2 weeks - 10 years |
| Incidence or rate of distant recurrence/metastasis | 44 (30) | 12 months - 10 years |
| Overall survival or mortality | 38 (26) | 30 days - 10 years |
| Disease- or progression-free survival | 26 (18) | 12 months - 10 years |
| Breast-cancer specific survival or mortality | 14 (9) | 12 months – 10 years |

^a^ Some studies reported more than one long-term oncological outcome.

**Table S5.** Surgical complications

| **Outcome category** | **Subdomain** | **Outcomes** |
| --- | --- | --- |
| Short term complications | Systemic | Thromboembolic complications (deep vein thrombosis/ pulmonary embolism)  Death  Cardiovascular complications (myocardial infarction)  Cerebrovascular complications (stroke)  Respiratory complications (pneumonia, reintubation)  Infection (organ/space/urinary, sepsis, septic shock)  Renal (renal failure) |
|  | Bleeding-related | Haematoma  Haemorrhage  Blood transfusion |
|  | Wound-related | Delayed healing  Wound dehiscence  Surgical site infection  Skin necrosis  Radiation-induced skin changes  Nipple-areola complex necrosis  Fat necrosis  Seroma |
|  | Major complications | Need for readmission  Need for reoperation |
| Long term complications | Wound-related | Dog ears  Hypertrophic scarring  Hypopigmentation |
| Patient reported symptoms | Breast symptoms | Altered sensitivity of breast/ nipple areolar complex  Breast fibrosis  Swelling in/around breast  Breast discomfort  Tightness/pulling |
|  | Arm and shoulder symptoms | Limitation of movement  Lymphedema  Shoulder pain  Weakness  Swelling under arm |
